# Generative AI and Language Models in Human Genetics and Health: From Variant Interpretation to Clinical Decision Support

**DOI:** 10.3390/genes17060723

**Published:** 2026-06-22

**Authors:** Yael Pinchevsky Itan, Yuval Itan

**Affiliations:** 1 The Windreich Department of Artificial Intelligence and Human Health, Icahn School of Medicine at Mount Sinai, New York, NY 10029, USA; 2 The Charles Bronfman Institute for Personalized Medicine, Icahn School of Medicine at Mount Sinai, New York, NY 10029, USA; 3 Department of Genetics and Genomic Sciences, Icahn School of Medicine at Mount Sinai, New York, NY 10029, USA; 4 Mindich Child Health and Development Institute, Icahn School of Medicine at Mount Sinai, New York, NY 10029, USA

**Keywords:** generative artificial intelligence, large language models, genomic language models, clinical genomics, variant interpretation, electronic health records, synthetic health data, protein design, retrieval-augmented generation, clinical decision support

## Abstract

Generative artificial intelligence (AI) is transforming biological and medical research and data analysis. Beyond analyzing existing information, these models can learn complex patterns and generate new data such as realistic protein sequences, genetic variants, or clinical notes. In molecular biology, language-like sequence models can read and generate DNA, RNA, and amino acid sequences to predict genetic variant effects, design new proteins, and explore molecular functions. In medicine, large language models (LLMs) trained on biomedical literature and electronic health records (EHRs) can summarize clinical findings, identify patterns, and provide decision support for clinicians and healthcare providers. Additionally, synthetic data generation can help protect patient privacy and augment existing disease datasets. While these advances make tasks that were previously impractical possible at scale, they also carry major risks, including producing convincing but incorrect results, reflecting hidden biases in the training data, and underperforming when real-world conditions change.

## 1. Introduction

Generative artificial intelligence (AI) generates new content by learning statistical patterns from existing data. Large language models (LLMs) are generally transformer-based generative AI models trained to predict the next token in a sequence based on the preceding context, while earlier sequence models included recurrent neural networks such as long short-term memory (LSTM) and gated recurrent unit (GRU) models. In genetics, these tokens are often nucleotides, k-mers, amino acids, or other sequence units, while in clinical text, they are usually words or subword units. Other generative AI families are generative adversarial networks (GANs), diffusion models (DMs), and protein structure-based generators. GANs use two neural networks where one makes fake examples and the other attempts to spot the fakes, so over time the generator learns to produce realistic synthetic data. DMs instead start from random noise and gradually clean it up to generate new synthetic examples that resemble the training data. Protein structure-based generators work directly on 3D protein shapes to suggest matching sequences or structures [1,2,3,4]. All these systems learn complex dependencies in the data that can then be used for specialized tasks. Language models can vary greatly in size and scope. Here, we use language models as a broad term for sequence models in DNA, RNA, protein, biomedical text, and clinical text, while LLMs refer to larger language models trained on broader datasets beyond these specific contexts.

**Figure 1:**
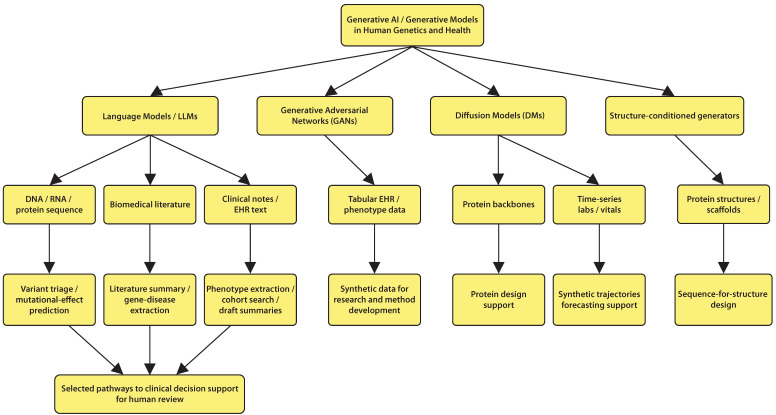
Generative AI model families and representative use cases in human genetics and health. The tree is organized from broad model families to data or input types and selected downstream applications. Representative applications include sequence modeling for variant interpretation, biomedical and EHR text analysis, synthetic EHR/phenotype and time series data generation, protein design, and selected pathways to clinical decision support for human expert review.

In this mini review, we use the term generative models (GMs) to refer to model families that can generate or transform data, including LLMs, GANs, diffusion models, and structure-based generators. These model families differ in architecture, input data, outputs, validations and clinical readiness. We therefore distinguish between current practical uses such as prioritization and summarization, and more speculative future uses such as autonomous interpretation or broad clinical decision making. We use the term generative AI more broadly to describe the applications and workflows built around these models [2,5]. Figure 1 provides a simplified overview of these model families and their main current applications in human genetics and health.

Human genetics and health can benefit from generative AI and LLMs because many core data types in genetics and medicine are sequences and unstructured text. DNA and protein sequences contain complex signals that are hard to decipher with rigid pre-defined rules, while clinical notes and scientific literature are mostly unstructured and high-volume [1,6]. In defined settings, GMs can learn task-specific representations that may support variant effect prediction, protein function and design, phenotype extraction, cohort retrieval, and concise clinical summaries [2,7,8,9].

Here we outline the following path for generative AI and LLMs in human genetics and health, from sequences through biomedical text/EHR to clinical decision support (CDS). This sequence-to-text-to-CDS path is used here as a practical framework, not as a claim that all these methods are equally mature or clinically validated. Across the sections of this overview, we emphasize differences in model maturity, validation, interpretability, and clinical readiness, distinguishing current uses such as prioritization and summarization from more speculative uses such as autonomous interpretation of data. This review covers: (1) DNA and protein sequence models, covering LLMs and non-LM generators (GANs, diffusion, structure-based) and how they support genetic variant interpretation; (2) biomedical and clinical text LLMs and synthetic data for supporting privacy and class balance; (3) interactive uses in research and clinician-patient communication; and (4) early steps toward CDS and what is needed for safe integration. Table 1 summarizes the methods discussed in this review by model family, primary task, and representative software for each section.

## 2. Sequence Models for Human Genomics

### 2.1. DNA Language Models

DNA language models (LMs) treat the genome as text, using k-mer (short overlapping sequence chunks) or other sequence tokens. Representative DNA LMs include DNABERT [10], which tokenizes genomic sequence into k-mers for shorter windows, and long-context genomic models such as Enformer [7], a hybrid convolutional-transformer model that predicts functional genomics and gene expression signals from long input windows, and Evo [11], which models very long genomic context for sequence understanding and generation. These methods can learn local motifs (such as promoters, enhancers, and splice sites) and longer dependencies across kilobases of nucleotides, and capture dependencies between regulatory regions and their target genes when given sufficiently long DNA sequence inputs. DNA LMs can help prioritize noncoding variants and better understand how gene activity differs between cell types, especially when fine-tuned on cell-type-specific data. Related genomic and epigenomic language models can also predict regulatory signals, including chromatin accessibility, histone-mark-associated features, and expression-related outputs from DNA sequence [7,12,13].

A major potential of DNA LMs is in variant regulatory effect prediction. Given a reference and an alternate allele, the model estimates changes in chromatin features or expression levels, which can help with the identification of relevant noncoding variants near known disease genes [7]. Related sequence-based splicing prediction models can also be used to score splice-altering variants by modeling exon-intron boundaries and local splice-regulatory signals [14]. For example, after standard filtering of a rare disease patient’s genome, a DNA LM can be used to compare the reference and alternative sequence around a candidate noncoding variant. Variants predicted to alter regulatory- or splice-related signals near a gene that is relevant to the patient’s phenotype can then be prioritized for further review and analyses. Therefore, these models are best used as a triage layer rather than as standalone diagnostic evidence. In benchmarks, these sequence-based models have shown useful performance for splice effect prediction, and long context regulatory models have performed well on chromatin accessibility prediction tasks, including DNase-seq or ATAC-seq assays in specific blood-derived cell lines [7,13,15]. However, the reported performance depends on the assay, tissue or cell type, benchmark dataset, and comparison method.

The limitations of DNA LMs include potential training data bias (specific assay, tissue and genetic ancestry representation) and uneven noncoding coverage (not all 3D or cell/tissue-specific interactions are captured). Learned embeddings and attention patterns may suggest biological signals, but they are not automatically interpretable mechanisms. Moreover, many genomic models are trained on imperfect reference genomes and on datasets that may over- or under-represent specific ancestries, assays and cell types. These limitations can be partially mitigated by stating the training/assay used, reporting the specific context window (the input sequence length), avoiding over-interpretation, and validating top predictions with experimental assays when possible [1,2,12,16,17]. The advantage of DNA LMs over traditional bioinformatics tools is therefore use case-dependent and should be assessed against task-specific benchmarks. Additional limitations of DNA LMs include incomplete modeling of epigenetic regulation, chromatin organization, tissue specificity, environmental effects, and post-translational modifications for downstream protein consequences.

### 2.2. Protein Language Models

Protein LMs train on amino acid sequences and learn statistical protein sequence patterns that often correlate with protein structure and function. Representative protein sequence LMs, which model large protein sequences and generate or score novel protein variants, include ProGen [18] and ProtGPT2 [19]. These models can capture residue variation, secondary structure patterns, active-site motifs, and mutational tolerance, and can help estimate missense variant impact, including effects that correlate with stability or function, enhancing variant prioritization for disease causality, and supporting sequence design toward desired protein functions [18,19,20].

An example of protein LM usage is de novo sequence proposals that are then filtered or optimized for a target fold (for example using in silico structure prediction) or for a motif-constrained scaffold, where some candidates are predicted to be plausible in silico and must then be experimentally tested for folding, stability, expression, binding, or activity [18,19]. In variant analyses, such model scores often correlate with deep mutational scanning (systematic assays that test the effects of many single amino acid changes in parallel) across several protein families, making them useful for broader pathogenicity assessments [20]. However, these correlations do not guarantee an accurate clinical pathogenicity prediction or successful protein design in a new biological context.

Real-world performance and reproducibility of such models are often limited because high in silico sequence likelihood does not necessarily predict empirical folding, stability, solubility, expression, localization, binding, or biological activity. High-likelihood predicted sequences can underperform for folding, stability, solubility, expression, localization, or binding. Training biases toward well-studied protein families or specific organisms/assays can also limit performance. To address these limitations, it is helpful to combine structural information when available, add evolutionary/conservation data, and test focused protein libraries rather than single designs [21,22,23].

### 2.3. Structure and Diffusion Generators (Non-LM)

Several generative AI models used in protein design are not classical next-token LMs. Representative methods for structure and diffusion generation include RFdiffusion [4], which generates de novo protein backbones or complexes, and ProteinMPNN [24], which performs structure-conditioned sequence-for-backbone design. Additional approaches include Chroma [25], ESM-IF1 [20,26], and EvoDiff [27], AlphaFold3 [21,28] and ESMFold [8] are not true generative AI systems in this context, but they are widely used to test and rank generated protein designs by estimating whether a proposed sequence folds into the intended protein structure.

An example of a non-LM workflow includes generating a new backbone with a diffusion model such as RFdiffusion [4], then designing protein sequences to fit it with ProteinMPNN [24], and finally selecting leading candidates with structure predictors where a subset may show measurable binding or activity in vitro.

It is important to note that wet lab validation is recommended (as with most computational predictions). The described predictions can miss protein misfolding, aggregation, off-target binding and more [22].

To summarize the current state of human genomics sequence models, DNA LMs are useful for regulatory/splice variant triage, protein LMs are helpful for mutational effects and protein designs, while non-LM generators enable structure-aware design and hypothesis generation [4,7,10,18,19].

## 3. Biomedical and Clinical Text Language Models

Text LMs operate on unstructured biomedical literature and EHR notes. Representative biomedical text LMs include BioGPT [6] which is trained on PubMed articles, and clinical question-answering (QA) models such as Med-PaLM [29] which are trained primarily on medical QA data and then further adapted for clinical use. The goal of these models is to compress long, unstructured text into concise, structured outputs that make it easier to review the literature or EHR notes and run analyses on the data [5,30]. In addition to these domain-specific methods, general-purpose chat assistants that include ChatGPT, Gemini, and Claude are widely used in research settings for literature review and summary, code generation, and exploratory clinical reasoning; because their outputs can vary across similar prompts even when using the same model version, studies that evaluate or use these tools in reproducible workflows should report the specific model version, access date, and prompting strategy. Many institutional tools access these models through application programming interfaces (APIs), often with some built-in security and privacy elements. Open-source models may improve transparency and local validation, whereas proprietary systems such as ChatGPT, Gemini, and Claude can be harder to test or reproduce because their model versions, training details, and inference pipelines may not be fully accessible. Fine-tuning on biomedical or local clinical data can improve performance, but overly narrow local adaptation may significantly reduce the broader capabilities of the model (a process that is often named “catastrophic forgetting”).

There are several tasks that can be performed by text LMs: summarization by condensing scientific papers or patients’ medical charts into problem lists and key findings; cohort retrieval, which maps inclusion/exclusion criteria to find relevant individual candidates; and gene–disease extraction to link genes, variants, phenotypes, and diagnoses into structured fields for searching and counting. Variant explainers can transform raw genomic annotations into a few bullet points that summarize the findings, sources and unknowns. For example, a table containing gene name, transcript, variant consequence, zygosity, inheritance model, population frequency, ClinVar/OMIM information, prediction scores, and patient HPO terms can be converted into a summary with references. This summary can state why the gene is relevant to the phenotype, whether the inheritance and zygosity fit the disease model, whether the variant is sufficiently rare for the specific phenotype, and what evidence remains uncertain or missing. Gene panel drafting by text LMs can utilize Human Phenotype Ontology (HPO) [31] terms to propose a gene list with brief rationale per gene and inclusion/exclusion flags. HPO symptom expansion can pull phenotypes from medical notes and add relevant related HPO terms to improve search and future analyses of the patient’s data. Technical lab test results can be translated into plain language with recommendations for next steps. CDS can direct clinicians and care providers to the exact guideline text across a broad spectrum of genomics and health topics [5,6,29].

An example is a genetics consultant who uploads a patient’s clinic notes, a pathology text, and a lab summary. The model returns: (i) the patient’s problem list, (ii) HPO-coded phenotypes of the patient with sources to back up the assignments/predictions, and (iii) any genetic variants in the records. The model may flag that the patient could meet criteria for consideration of an inherited cardiometabolic panel and cite the relevant sources to support the clinician’s downstream review and decision-making process [5]. In practice, this requires de-identification and/or institution-approved secure deployment of the workflow.

When sharing patient notes is restricted, text LMs can be used to generate synthetic clinical text that approximates the source data, which may reduce re-identification risk, and can help augment datasets with limited data such as rare diseases [32].

Risks of text LMs include hallucinations, hidden biases, and stale or site-specific knowledge that cannot be properly generalized. To partly mitigate this, data should be based on trusted information, legitimate citations, and include expert review in the training and validation process [33,34,35].

In summary, text LMs can aid in retrieval, citation, and human-like review of literature and EHR unstructured free-text notes into compact and useful entries for health and genetics workflows [5,6,29].

## 4. Synthetic Data for Genomics and Clinical Research

Synthetic data enables data analysis and method development while protecting patients’ privacy and mitigating class imbalance (when the sample sizes of some patient groups, controls, or outcomes are very different or too small for model training). Privacy restrictions on accessing EHR data can limit both data sharing and the analysis of existing data. Moreover, disease cohorts contain more information on common conditions and majority population groups, while data on rare diseases and minority populations often do not have sufficient statistical power. Synthetic cohorts can help address these issues by generating realistic, high-quality, patient-like information without live records access concerns [3,36,37]. However, synthetic data are not automatically anonymous and should still be evaluated for privacy leakage, memorization, and membership inference risk, as well as how well they reflect the real data before sharing or downstream usage. In genomics, synthetic data may also distort rare variant frequencies, linkage disequilibrium, population stratification, and genotype–phenotype correlations. In EHR data, synthetic generators may also fail to preserve complex relationships among diagnoses, medications, laboratory values, procedures, and clinical outcomes. These can compromise downstream analyses if not checked against real held-out data.

Methods for synthetic data generation include GANs and related deep generators for creating tabular EHR snapshots (tables of patient data), time-series models for physiologic signals and lab tests (such as heart rate and various lab panels), and LMs for short phenotype summaries that are conditioned on structured fields, with outputs as simple tables (comma or tab-separated) [3,36,37]. Representative tabular EHR GANs, which learn joint distributions of variables in structured patient records, include medWGAN and medBGAN [3]. For patient genomics data, tabular generators can synthesize variant-level predictions with phenotype codes. Diffusion-based time-series models such as TimeGrad (where TimeGAN is often used as a reference baseline) [38] can sample plausible trajectories for patient vitals and labs over days or weeks, supporting probabilistic forecasting and simulation.

A usage example is building a rare immunodeficiency machine learning (ML) classifier when only a few dozen labeled cases are available, a training set that is too small for an effective model. To resolve this, a tabular data generator is trained on an entire immunodeficiency clinic dataset, learning patterns from real patients. The generator is then conditioned on immunodeficiency phenotype codes to create synthetic records that approximate real test results and medications that resemble those of true immunodeficiency cases. These synthetic records can be used to choose features and tune hyperparameters for the ML classifier. Finally, the classifier is trained and evaluated on real, held-out patients [3,36].

## 5. Clinical Decision Support and Integration

Clinical decision support (CDS) should be narrow, source-linked, and easy for clinicians and healthcare providers to review. Multimodal pipelines combine genomics (variants and gene panels), text (clinical problem lists and HPO terms), and labs (key values and trends), and some systems also add relevant images when needed. Domain LMs (e.g., BioGPT) and clinical QA models (e.g., Med-PaLM) are used for limited, well-defined clinical tasks such as drafting a short note, generating a patient checklist, or pointing to the relevant guideline paragraph, while clinicians make the final decisions [5,6,29,30]. Many early CDS prototypes and commercial tools rely on general-purpose LLMs such as ChatGPT, Gemini, or Claude as back-end engines, often including institution-specific guardrails; given the inherent output variability of these LLMs even when using similar prompts, the model, prompt, and local configuration should be documented. Clinical deployment also requires local EHR interoperability, privacy controls, liability planning, and compliance with frameworks such as HIPAA, FDA oversight and CE-marking requirements where relevant, and institutional AI governance.

Pilot clinical deployments should start in settings where the benefit is clear and the risk is low, such as pre-visit chart summaries for genetics clinics (short overviews of a patient’s history prepared before the visit), variant re-reviews (lists of genetic variants that need to be reassessed when new evidence appears), and phenotype summaries for case conferences (concise descriptions of the patient’s key signs, symptoms, and test findings used in team discussions). In these use cases, LMs can assist by drafting short, structured notes that point clinicians to relevant evidence. One practical way to make these outputs linked to their source data is retrieval-augmented generation (RAG), which first retrieves relevant information from curated local sources, such as institutional standard operating procedures, reference databases, variant knowledgebases, and clinical guidelines, and then uses this material to draft a grounded answer. In EHR and clinical genomics workflows, RAG can reduce unsupported statements, but it does not eliminate errors when retrieval is incomplete, outdated, or incorrectly summarized [5,29]. For example, a genetics consultant can query Med-PaLM with HPO-coded symptoms and relevant guidelines to draft a 5–7-line note, then ground each line using RAG-derived citations [29,34]. Clinical genomics outputs should include uncertainty, confidence, or calibration information when available, and should link each statement to the underlying variant, gene, phenotype, guideline, or database source.

## 6. Limits and What’s Next

The tools presented can be helpful for a wide range of applications, but they also have major limitations and risks. Required performance of models depends on their intended use: lower-performing models may still be helpful with low-risk triage, whereas diagnostic variant classification, treatment recommendations, reproductive counseling, or automated matching to high-risk interventions require very high performance, careful calibration, and prospective validation, potentially including metrics such as Matthews correlation coefficient above 0.9 for some tasks. Major risks include hallucinations, where models can invent facts or distort values when input is limited. In genomics and rare disease diagnostics, such errors are especially dangerous because an incorrect gene–disease link, inheritance statement, or variant interpretation can substantially affect testing, counseling, treatment, or family screening. Other risks include bias in training data that over-represent certain tissues, ancestries, and well-studied genes; distribution shifts as documentation style, lab platforms, and local variant pipelines change over time; uneven benchmarks and test sets that differ across papers; and synthetic-data fidelity risks, where generators can miss edge cases or leak patterns of real patients. Because these factors can evolve over time, deployed models can become stale or unreliable without periodic revalidation on up-to-date data. Models that perform well on curated benchmarks may still fail in noisier real-world EHR data, under-represented populations, rare phenotypes, or new biological contexts. These issues are especially relevant when informally consulting general-purpose chatbots without institutional guardrails. In summary, synthetic data should not replace real-world evaluation [9,33,34,35,36]. Additional concerns include computational cost, GPU requirements, energy use, informed consent, genomic data ownership, genetic discrimination, algorithmic fairness, and possible misuse such as synthetic pathogenic sequence design or misleading biomedical content. Privacy-preserving approaches, including federated learning and local hospital deployment, may reduce some risks of data sharing, but they do not remove the need for validation, privacy review, and ongoing data monitoring. As clinical AI systems evolve, governance frameworks will also need to be updated to address changing model capabilities, deployment settings, clinician oversight, and accountability.

Future developments in the field may include unified multimodal models that handle genomics, imaging, proteomics, clinical notes, and laboratory data in one pipeline, smaller on-site models that keep patients’ data more secure, clearer validation and interpretation in patient genetics data integrated with EHR data, and agentic systems that coordinate limited tasks such as cohort search, clinical trial matching, variant re-evaluation, and checklist drafting for human review [2,5].

Agentic AI refers to systems that coordinate multiple steps toward a defined goal such as data extraction, eligibility checking, and summary drafting. In clinical genetics, near-term uses may include cohort search, clinical trial matching, variant re-evaluation, and checklist drafting for clinician review. A practical path forward is to use narrow, well-defined scopes, high-quality training sets, trusted data retrieval, human review, and ongoing accuracy measurements. Near-term priorities include external validation, clearer reporting of uncertainties, safer use of patient data, and prospective testing in defined clinical workflows. The main clinical value reviewed here is likely decision support, whereas decision making remains the responsibility of clinicians and healthcare providers [1,5,34].

## Figures and Tables

**Table 1:** A summary of the methods referenced in this review by model family, primary task, and representative software.

Data Type/Model Family	Primary Task	Representative Method(s)	Main Limitation	Clinical Readiness
DNA sequence LMs	Regulatory/splice and noncoding variant scoring	DNABERT, Enformer	Tissue/context bias; limited interpretability	Triage/research support
Protein sequence LMs	Missense/functional effect signals; sequence seeds for protein design	ProtGPT2, ProGen	In silico scores may not translate to function	Research/variant support
Structure prediction and diffusion generators	Structure and interaction prediction, Protein design	AlphaFold 3, RFdiffusion, ProteinMPNN	Requires wet lab validation	Research/design, drug discovery
Biomedical and clinical text LMs	Summaries, cohort search, gene–disease extraction, clinical text	BioGPT, Med-PaLM	Hallucination; site-specific bias	Drafting/review support
Synthetic data, tabular	Phenotype/genomics-adjacent table generation	medWGAN, medBGAN	Privacy leakage; may distort real-data patterns	Research/method development
Synthetic data, time series	Synthetic vitals/labs over time; predict next values/events	TimeGrad	May miss rare trajectories	Research/simulation

## Data Availability

No new data were created or analyzed in this study. Data sharing is not applicable to this article.
